# Squamous cell carcinoma of the thyroid gland in an elderly female presenting as a rapidly enlarging thyroid mass

**DOI:** 10.1016/j.ijscr.2020.04.064

**Published:** 2020-05-11

**Authors:** Ramadhan T. Othman, Azad Mustafa Ahmed Baizeed, Ayad Ahmad Mohammed

**Affiliations:** a Department of Internal Medicine, College of Medicine, University of Duhok, Kurdistan Region, Iraq; b Department of Dental Basic Sciences, University of Duhok, Kurdistan Region, Iraq; c Department of Surgery, College of Medicine, University of Duhok, Kurdistan Region, Iraq

**Keywords:** Squamous cell carcinoma, Thyroid cancer, Neck mass, Thyroid, Radiotherapy

## Abstract

**Background:**

Squamous cell carcinoma of the thyroid gland is an extremely rare tumor with a very aggressive clinical behavior and poor prognosis. The tumor may arise as a primary tumor within the thyroid gland or as a component of anaplastic or undifferentiated thyroid tumors.

**Case presentation:**

A 70-year-old lady with history of long standing multinodular goiter presented with progressive enlargement of a midline nodule for 3 months which was associated with dyspnea and dry cough. The mass was hard and fixed. The voice was normal, other parts of the general examination were unremarkable. Fine needle aspiration was done for the mass which revealed malignant cells mixed with inflammatory cells. During surgery there was a hard and fixed mass arising from the isthmus of the thyroid gland that was locally invading, complete excision was not possible, debulking was done. Histopathology showed moderately differentiated squamous cell carcinoma of the thyroid gland. The tumor underwent local progression 6 months later and the patient was sent for radiotherapy.

**Conclusion:**

When thyroid tumor is advanced at time of diagnosis, radiotherapy is the main form of treatment which may induce reduction in the size of the tumor and decrease pain, radiotherapy may also be given on neoadjuvant bases which may render resection possible in some patients. The tumor is usually not responsive to chemotherapy. The overall survival is uniformly poor regardless of the primary form of treatment, the median survival of the patients from the time of diagnosis is few months in most cases.

## Highlights

• Squamous cell carcinoma of the thyroid gland is an extremely rare tumor.
• It may be primary tumor within the thyroid gland or as a component of anaplastic or undifferentiated tumors.
• The tumor has a very aggressive clinical behavior with a median survival of few months.

## 1 Introduction

Squamous cell carcinoma of the thyroid gland is an extremely rare tumor with a very aggressive clinical behavior and poor prognosis. Worldwide, few cases are reported with a reported incidence of less than 1% from all thyroid tumors [1,2].

The tumor may arise as a primary tumor within the thyroid gland or as a component of anaplastic or undifferentiated thyroid tumors. The presence of squamous cell inside the thyroid gland is reported by many authors and is thought to present as a result of metaplasia of normal follicular epithelial cells [3].

In some cases, squamous cell carcinoma of the thyroid gland may be associated with other types of well differentiated thyroid tumors, in such cases it is very difficult to conclude whether the tumor is arising de novo or as a result of a change in the histopathological characteristics of the well differentiated thyroid cancers [4].

The disease usually affects the elderly people, and most cases have an advanced disease at the time of presentation with invasion to the trachea, esophagus, and the vessels in the region of the neck, there may be distant metastasis at the time of the first presentation [1,5].

Patients usually present with a rapidly enlarging neck mass, which may be painful. Most patients have symptoms of airway obstruction like dyspnea and stridor [6].

Once this type of tumor is diagnosed, the possibility of local extension to the larynx or pharynx, or the possibility of metastatic tumor should be excluded [2].

The work of this report case has been reported in line with the SCARE 2018 criteria [7].

## 2 Patient information

A 70-year-old lady with history of long standing multinodular goiter presented with progressive rapid enlargement of a midline thyroid nodule for 3 months which was associated with dyspnea and dry cough.

### 2.1 Clinical findings

During examination there was multinodular goiter with a hard and fixed mass in the midline of the neck, the mass was moving with swallowing, the voice was normal and the patient was sent for vocal cords evaluation by flexible laryngoscopy, which showed no evidence vocal cord paralysis. Other parts of the general examination were unremarkable.

### 2.2 Diagnostic assessment

The complete blood count was normal, and the thyroid function test was also normal.

FNA was performed and confirmed malignant cells mixed with inflammatory cells.

Preoperative echocardiography showed good cardiac function and preoperative workup including CXR showed no evidence of any lung lesion during the first operation.

### 2.3 Therapeutic intervention

During surgery there was a hard and fixed mass arising from the isthmus of the thyroid gland with multinodular thyroid enlargement, the mass was about 7 × 7 cm and was locally invading the larynx and the carotid sheath, complete excision was not possible because of unclear anatomical plans, debulking surgery was done and the sample was sent for the histopathology. A suction drain was placed in the neck at the site of resection which was removed after 3 days.

**Fig. 1:**
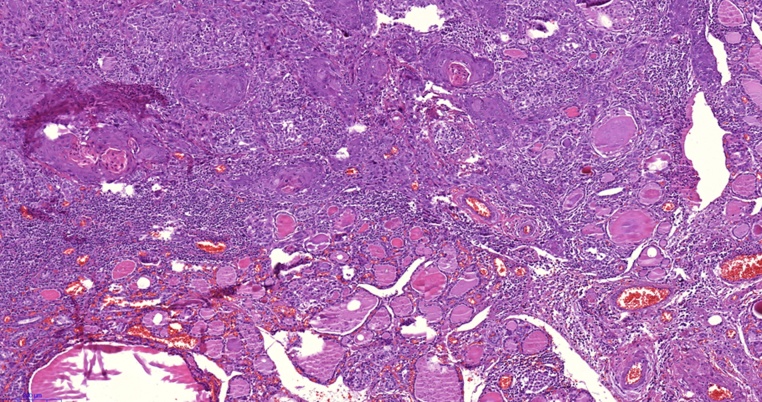
A microscopic picture (X100) showing malignant well differentiated squamous components in the upper half of the image that infiltrate the thyroid tissue which is shown below.

**Fig. 2:**
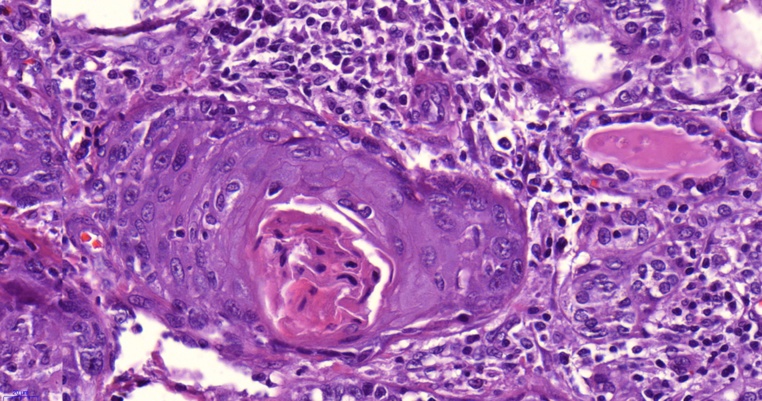
A microscopic picture (X400) showing malignant well differentiated squamous components containing central keratin material.

**Fig. 3:**
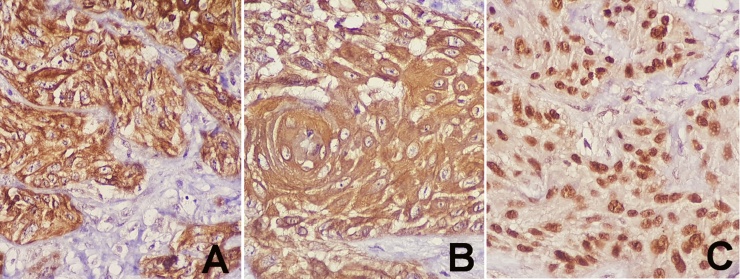
Microscopic pictures demonstrating positive immunohistochemical staining: (A) Positive cytoplasmic staining for Cytokeratin AE1/AE3, (B) Positive cytoplasmic staining for CK5/6, (C) Positive nuclear staining for P63.

**Fig. 4:**
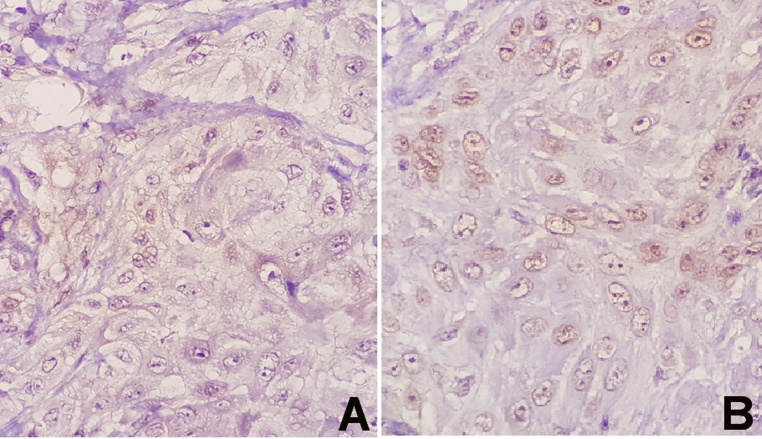
Microscopic pictures demonstrating negative immunohistochemical staining: (A) Negative cytoplasmic staining for thyroglobulin, (B) Negative cytoplasmic staining for TTF-1.

The final histopathological result was consistent with mixture of thyroid nodules and moderately differentiated squamous cell carcinoma. Fig. 1, Fig. 2, Fig. 3, Fig. 4.

### 2.4 Follow-up and outcomes

6 months after surgery the patient presented with progression of the mass at the same site, which was hard and fixed, associated with multiple enlarged hard cervical lymph nodes at both sides of the neck. The patient was sent for CT-scan which showed an evidence of an ill-defined heterogeneous mass at the site of surgery and left lobe of thyroid, the mass was invading the esophagus and the trachea with enlarged multiple bilateral cervical lymph nodes. There was evidence of 2 possible metastatic nodules in the upper lobe of right lung. The patient was sent for post-operative radiotherapy.

## 3 Discussion

The normal thyroid gland doesn’t contain squamous cells except in some very rare occasions such as the presence of some embryonic elements or in cases of chronic inflammatory conditions affecting the thyroid gland. Examination of the tumors under electron microscope usually shows the absence of keratohyline within the squamous cells suggesting that the cell of origin may be an altered thyroid follicular epithelial cells [2,3].

There are three possible theories explaining the occurrence of squamous cell carcinoma within the thyroid gland. The first theory is the embryonic nest theory, in which the authors suggest that the squamous cells are originated from embryonic remnants like thyroglossal duct, thymic epithelium or from the ultimo-bronchial body, the second theory is the metaplasia theory; in this theory the authors suggest that environmental stimuli such as chronic inflammation or Hashimoto’s thyroiditis result in squamous metaplasia of the thyroid follicular epithelium, the third theory is the de-differentiation theory; in which the authors suggest that an thyroid cancer de-differentiate into squamous cell cancer [2].

The differential diagnoses of squamous cell carcinoma of the thyroid gland are cancer metastasizing of extending to the thyroid gland from the larynx, esophagus, nasopharynx, and the lungs. Although metastatic squamous cell carcinoma to the thyroid gland is very rare, but in most of the cases it is possible to differentiate primary from secondary tumors on the basis of clinical examination, endoscopic findings, imaging and histopathological evaluation [3,8,9].

Whenever possible, complete surgical resection with post-operative radiotherapy is the best management option, patients should be observed closely after surgery to detect recurrence early [5].

Transient palliation is achieved by partial resection followed by radiotherapy, but when the tumor is not excised completely, it regrows again within few months in the majority [5].

When the tumor is advanced at the time of diagnosis, radiotherapy is the main form of treatment which may induce reduction in the size of the tumor and decrease pain, radiotherapy may also be given on neoadjuvant bases which may render resection possible in some patients. The tumor is usually not responsive to chemotherapy [1].

The overall survival for such tumors is uniformly poor regardless of the primary form of treatment, the median survival of the patients from the time of diagnosis is few months in most cases [8]

When the facility and the expertise are available, extensive and radical surgery may be performed especially when the prognosis is promising and the patient is young. This may involve total thyroid resection followed by radical cervical lymph node resection and resection of the involved muscles and soft tissues, the tissue defects may be then reconstructed using different types of muscle flaps [10].

## 4 Conclusion

When the tumor is advanced at the time of diagnosis, radiotherapy is the main form of treatment which may induce reduction in the size of the tumor and decrease pain, radiotherapy may also be given on neoadjuvant bases which may render resection possible in some patients. The tumor is usually not responsive to chemotherapy. The overall survival is uniformly poor regardless of the primary form of treatment, the median survival of the patients from the time of diagnosis is few months in most cases.

## Patient’s perspective

I was informed that there is a tumor in my thyroid gland, I am ready for any form of treatment in the future.

## Informed consent

An informed written consent was taken from the patient for reporting the case and the accompanying images.

## Declaration of Competing Interest

There is no any conflict of interest.

## Sources of funding

The authors are the main source of funding.

## Provenance and peer review

Not commissioned, externally peer-reviewed

## Ethical approval

Ethical approval has been exempted by my institution for reporting this case.

## Consent

An informed written consent was taken from the patient for reporting the case and the accompanying images.

## Author contribution

The concept of reporting the case, data recording, and drafting the work done by Dr Ramadhan T Othman and Dr Ayad Ahmad Mohammed.

Dr Ramadhan T Othman took the consent from the patient for publishing the case. Final approval of the work to be published was done by Dr Ayad Ahmad Mohammed.

## Registration of research studies

This work is case report and there is no need of registration.

## Guarantor

Dr Ayad Ahmad Mohammed is guarantor for the work.
